# Mediation Role of Physical Fitness and Its Components on the Association Between Distribution-Related Fat Indicators and Adolescents’ Cognitive Performance: Exploring the Influence of School Vulnerability. The Cogni-Action Project

**DOI:** 10.3389/fnbeh.2021.746197

**Published:** 2021-09-08

**Authors:** Sam Hernández-Jaña, Javier Sanchez-Martinez, Patricio Solis-Urra, Irene Esteban-Cornejo, Jose Castro-Piñero, Kabir P. Sadarangani, Nicolas Aguilar-Farias, Gerson Ferrari, Carlos Cristi-Montero

**Affiliations:** ^1^Physical Education School, Pontificia Universidad Católica de Valparaíso, Valparaíso, Chile; ^2^Escuela de Kinesiología, Facultad de Salud, Universidad Santo Tomás, Viña del Mar, Chile; ^3^PROFITH “PROmoting FITness and Health Through Physical Activity” Research Group, Sport and Health University Research Institute (iMUDS), Department of Physical Education and Sports, Faculty of Sport Sciences, University of Granada, Granada, Spain; ^4^Faculty of Education and Social Sciences, Universidad Andres Bello, Viña del Mar, Chile; ^5^GALENO Research Group, Department of Physical Education, Faculty of Education Sciences, University of Cádiz, Puerto Real, Spain; ^6^Biomedical Research and Innovation Institute of Cádiz (INiBICA) Research Unit, Cádiz, Spain; ^7^Universidad Autónoma de Chile, Providencia, Chile; ^8^Escuela de Kinesiología, Facultad de Salud y Odontología, Universidad Diego Portales, Santiago, Chile; ^9^Department of Physical Education, Sports and Recreation, Universidad de La Frontera, Temuco, Chile; ^10^Escuela de Ciencias de la Actividad Física, el Deporte y la Salud, Universidad de Santiago de Chile (USACH), Santiago, Chile

**Keywords:** cognition, physical activity, children, school, obesity, fatness, fat distribution

## Abstract

**Background**: Physical fitness and fatness converge simultaneously modulating cognitive skills, which in turn, are associated with children and adolescents’ socioeconomic background. However, both fitness components and fat mass localization are crucial for understanding its implication at the cognitive level.

**Objective**: This study aimed to determine the mediation role of a global physical fitness score and its components on the association between different fatness indicators related to fat distribution and adolescents’ cognitive performance, and simultaneously explore the influence of school vulnerability.

**Methods**: In this study, 1,196 Chilean adolescents participated (aged 10–14; 50.7% boys). Cardiorespiratory fitness (CRF), muscular fitness (MF), and speed-agility fitness (SAF) were evaluated, and a global fitness score (GFS) was computed adjusted for age and sex (CRF + MF + SAF *z*-scores). Body mass index *z*-score (BMIz), sum-of-4-skinfolds (4SKF), and waist-to-height ratio (WHtR) were used as non-specific, peripheral, and central adiposity indicators, respectively. A global cognitive score was computed based on eight tasks, and the school vulnerability index (SVI) was registered as high, mid or low. A total of 24 mediation analyses were performed according to two models, adjusted for sex and peak high velocity (Model 1), and adding the school vulnerability index (SVI) in Model 2. The significance level was set at *p* < 0.05.

**Results**: The fitness mediation role was different concerning the fatness indicators related to fat distribution analyzed. Even after controlling for SVI, CRF (22%), and SAF (29%), but not MF, mediated the association between BMIz and cognitive performance. Likewise, CRF, SAF and GFS, but not MF, mediated the association between WHtR and cognitive performance (38.6%, 31.9%, and 54.8%, respectively). No mediations were observed for 4SKF.

**Conclusion**: The negative association between fatness and cognitive performance is mitigated by the level of adolescents’ physical fitness, mainly CRF and SAF. This mediation role seems to be more consistent with a central fat indicator even in the presence of school vulnerability. Strategies promoting physical fitness would reduce the cognitive gap in children and adolescents related to obesity and school vulnerability.

## Introduction

An excess of body fat has been associated with a diversity of metabolic, cardiovascular, and mental health conditions (Halfon et al., 2013; Sahoo et al., 2015). Children and adolescents with overweight or obesity present lower cognitive functioning (Esteban-Cornejo et al., 2020) in several cognitive domains such as attention, executive functioning, memory, and visuospatial performance (Liang et al., 2014). An adequate cognitive development in childhood is crucial for both short and long-term, due to its future impact on predictors related to socioeconomic status, health, and behavior, which might counteract the adverse effect of children’s social environment (Feinstein and Bynner, 2004).

In this sense, social disadvantages and inequalities have been related to worse cognitive developments and obesity in childhood (Ruiz-Hermosa et al., 2019; Vazquez and Cubbin, 2020). For instance, children and adolescents living in a vulnerable context showed increased cortisol levels, reduced gray and white brain matter, lower performance in working memory, inhibitory control, and cognitive flexibility (Ursache and Noble, 2016b) and thereby, a lower performance in their general cognitive functioning (Hackman and Farah, 2009; Brito and Noble, 2014). Thus, both obesity and social vulnerability converge, affecting normal cognitive development in children and adolescents. This complex scenario might be mitigated in early stages such as adolescence when the brain defines its structure and functioning, and still it is possible to evoke behavioral changes (Herting and Chu, 2017; Stillman et al., 2020).

Many strategies have been suggested to improve children and adolescents’ cognitive performance (Diamond and Ling, 2016; Schoentgen et al., 2020). For this purpose, physical fitness seems to be an enjoyable and low-cost approach associated with reducing body fat mass and the influence of social vulnerability (Yang et al., 2007; Åberg et al., 2009). For instance, a study in children aged 5–7 years showed that physical fitness mediates the adverse relationship between body mass index and cognitive performance, and this outcome seems to be independent of socioeconomic vulnerability (Ruiz-Hermosa et al., 2019). Furthermore, a study using a structural equation model established that adolescents’ physical fitness mediates the relationship between body mass index and cognitive performance (Lemes et al., 2021). Nonetheless, school vulnerability presented an inverse association with cognitive performance, which was only partially mediated by physical fitness (Lemes et al., 2021). Therefore, current evidence suggests a positive influence of the physical fitness level in the association between fatness and cognitive functioning, which could even be maintained despite the detrimental impact of the children’s social background.

Despite this evidence, there are still gaps to cover in order to improve global understanding in this research area. Some authors point out that cardiorespiratory fitness (CRF) has been the most studied fitness indicator on cognition (Esteban-Cornejo et al., 2017; Kao et al., 2017), but scarce evidence has explored how other fitness components such as muscular fitness (MF) and speed-agility fitness (SAF) are related to cognitive functioning. Similarly, fatness indicators have been limited mainly to the use of the body mass index (Ruiz-Hermosa et al., 2019); nevertheless, evidence indicates that the location of fat mass is crucial. For instance, visceral fat seems to affect cognitive functioning to a greater extent than other general (non-specific) or peripheral indicators (Schwartz et al., 2013). Also, longitudinal and bidirectional evidence in children and adolescents have shown that fatness may play a more relevant role in the risk of developing metabolic syndrome compared with CRF (Reuter et al., 2021), and that fatness changes were associated with future CRF levels, independently of baseline CRF (Perez-Bey et al., 2020). The aforementioned, support our theoretical approach considering fatness as a predictor of cognitive performance.

Therefore, the present study aims to determine the mediator role of CRF, MF, SAF, and a global fitness score (GFS) on the association between fatness indicators related to fat distribution such as body mass index *z*-score (BMIz), sum-of-4-skinfolds (4SKF), and waist-to-height ratio (WHtR; non-specific, peripheral, and central adiposity indicators, respectively) and a global cognitive score in adolescents. Furthermore, this study explored the influence of the school vulnerability index (SVI) as a covariate. It is hypothesized that the GFS and all its components mitigate the inverse relationship between fatness indicators and cognitive performance and that mediation effects remain stable even when SVI is included in the model. At the same time, it is hypothesized that WHtR could be the most consistent fat indicator compared to BMIz and 4SKF, in the mediation role of fitness, due to its greater specificity.

## Materials and Methods

This study is part of the Cogni-Action Project, which determines the associations of physical activity, sedentary behavior, and physical fitness with brain structure and function, cognitive performance, and academic achievement in a large sample of Chilean adolescents (Solis-Urra et al., 2019). It was conducted from March 2017 to October 2019 and involved adolescents from the public, subsidized, and private schools in Valparaíso, Chile. The project was approved by the Ethics Committee of Pontificia Universidad Católica de Valparaíso (BIOEPUCV-H103-2016) and was registered in the Research Registry (ID: researchregistry5791). Written consents or assents were obtained before participation from corresponding school principals, parents, and adolescents. The present study was performed according to STROBE guidelines (Strengthening the Reporting of Observational Studies in Epidemiology) for cross-sectional studies (von Elm et al., 2008).

### Study Population

The sample size was calculated based on the total enrolment of children and adolescents between grades 5–8, according to the student universe (*n* = 951, 962) indicated by the Ministry of Education. More information about sample size estimation can be found elsewhere (Solis-Urra et al., 2019). Overall, a total of 797 participants were needed for representativeness, nonetheless, 1,296 adolescents (10–14 years old) from 19 schools participated in the project. Important to note, this project and study use the definition of adolescence which establishes it as the period between 10–24 years of age (Sawyer et al., 2018). Inclusion criteria for this project were girls and boys from grades 5–8, while the exclusion criteria for this study were incomplete fitness, fatness, cognitive and covariates data. Finally, 1,196 participants were included in this study.

### Measurements

Adolescents were evaluated at schools in two sessions of 4 h separated by 8 days. Cognitive performance and anthropometric measurements were assessed in the first session, whereas physical fitness was evaluated in the second session. Trained staff evaluated all variables; moreover, adolescents had familiarization trials before each test.

### Physical Fitness Assessment

The ALPHA-fitness test battery was used to evaluate three physical fitness components (CRF, MF, and SAF) by four different field-based tests. The validity and reliability of this battery have been described in previous research (Ruiz et al., 2011). It was suggested to wear suitable sportswear to perform tests in sport or indoor fields during the morning (between 9:30 and 12:00). Instructions were verbally provided, and each test was explained and demonstrated to ensure optimal performance. Adolescents practised the tests and performed them when they felt prepared to start.

### Global Fitness Score

To compute the GFS, physical fitness was assessed through the ALPHA fitness test battery, which evaluates three main fitness components, CRF, MF, and SAF (Ruiz et al., 2011). A *z*-score of each component was calculated adjusted for age and sex, and all three were added. The evaluations carried out are detailed below.

### Cardiorespiratory Fitness

The 20-m shuttle run test was used to evaluated CRF grouping between eight to 10 participants, and they were guided to the starting line. The run rhythm (pace) was indicated by a sound signal and started at 8.5 km/h, increasing 0.5 km/h every minute. They started the test from the starting line and had to run 20 meters to the second line and wait for the next signal to run back to the starting line. A physical education teacher ran beside the adolescents during the first 2 min to ensure the correct progression and adaptation to the test. The trial ended when participants could not keep the velocity of the test or failed to reach the line twice. Total time in seconds was registered as previously recommended (Tomkinson et al., 2019). Lastly, a *z*-score according to age and sex was created as a normalized CRF score.

### Muscular Fitness

The MF indicator was calculated according to upper and lower limb strength. The maximum handgrip strength was used to evaluate upper limb strength using a dynamometer (Jamar Plus+ Digital Hand Dynamometer, Sammons Preston, USA), previously adjusted for participants’ hand size (measures of 0–90 kg and 0.1 kg precision). The test was performed on both hands (twice) with a fully extended elbow in a standing position, registering the maximum score. A relative measure of upper limb strength was calculated, dividing the best score (both hands) by body weight.

The standing long jump test was used to determine the lower limb strength. Adolescents must stand with both feet in parallel behind the starting line. They had to jump on the verbal signal as far as possible with both feet simultaneously. They performed twice this test, resting 1 min between attempts, and the longest jump was recorded in centimetres. Lastly, sex- and age-specific *z*-score from the upper and lower limb tests were added to calculate the MF score.

### Speed-Agility Fitness

The 4 × 10 m shuttle run test was used to determine SAF, which involves the speed of movement, agility, and coordination. This test consists of running between two lines (5 m in width) separated by 10 m in length, with both lines having a cone placed as a point reference. Every participant had to run as fast as possible, and on reaching the first line, they had to grab a cloth located ~50 cm and run back, carrying it to the start line (this procedure was repeated three times). After that, they had to repeat the sequence until the ending of the test. Every adolescent had two opportunities, and the fastest time was recorded in seconds. Furthermore, time was multiplied by −1; a higher score means a better performance. Lastly, a *z*-score according to age and sex was created as a normalized SAF score.

### Cognitive Performance

Cognitive performance was evaluated using the NeuroCognitive Performance Test (NCPT) from Lumos Labs, Inc., which has demonstrated acceptable reliability and validity to assess cognitive performance (Morrison et al., 2015). This test based on a web-based platform allows measuring several cognitive domains such as working memory, visuospatial memory, psychomotor speed, fluid and logical reasoning, response inhibition, numerical calculation, and selective and divided attention.

The NCPT was taken in groups of 25 participants (each one had a laptop provided by the research team) in school classrooms, lasting roughly 1 h the entire session. First of all, the session’s objective was provided through a brief explanation, demonstration, practice, and execution before each test. Furthermore, any adolescents’ questions about the procedure were immediately answered by an instructor before starting each cognitive test. A summary of all cognitive tests is shown in Supplementary Figure 1, and more information about them can be found in previous research (Morrison et al., 2015; Solis-Urra et al., 2019). Finally, following the original battery procedure, each test was scaled according to a normal inverse transformation of the percentile rank (Morrison et al., 2015). Hence, it calculated a score derived on the same normal distribution with a mean and standard deviation of 100 and 15.

### Fatness Indicators

General and non-specific indicator of adiposity (BMIz): The height and weight were measured with a digital scale OMRON (HN-289-LA, Kyoto, Japan) with a precision of 0.1 kg and a portable stadiometer SECA (model 213, GmbH, Germany) with a precision of 0.1 cm, respectively. The World Health Organization, 2007 growth reference was used to determinate BMIz for school-age children (de Onis et al., 2007).

Peripheral adiposity indicator (4SKF): Triceps, biceps, subscapular, and suprailiac skinfolds were measured with a Slim Guide calliper (Creative Health Products, Plymouth, Michigan, United States) and added to calculate the 4SKF (Cristi-Montero et al., 2019).

Central adiposity indicator (WHtR): The minimum waist circumference was assessed with an inextensible tape (Lufkin, Apex, NC, Unites States), and then it was divided by height in centimetres to obtain WHtR (waist[cm]/height [cm]).

### Covariates

Sex, peak high velocity (PHV), and SVI were used as covariates. Sex has been considered a relevant moderator in this research area because visceral fatness, for instance, may impact more strongly in female subjects (Schwartz et al., 2013). Likewise, there is a significant interindividual variance in biological maturation timing among adolescents (Lloyd et al., 2014). Thereby, differences between chronological and biological age would be reflected in brain development or cognitive abilities (Brown et al., 2012). Hence, PHV was calculated as a maturity indicator (Moore et al., 2015), subtracting the PHV age from the chronological age. Differences among years were established as a maturity offset value.

Socioeconomic status is a potent predictor of diverse domains such as language skills, executive function, memory, and social-emotional processing (Ursache and Noble, 2016a). However, in Latin-American countries, school characteristics (i.e., economic, social, and cultural status) seem to be a stronger predictor of adolescents’ cognitive and school performance than socioeconomic status (Flores-Mendoza et al., 2015). SVI is an index to measure students’ socioeconomic vulnerability at public/subsidized funding schools. It involves the family’s socioeconomic status, the educational level of parents-tutors, student health condition, physical and emotional wellbeing, and school location (López et al., 2017). Then, schools were classified as low (<10), middle (≥10 to <60), and high (≥60) SVI. A value of zero is assigned to private schools.

### Statistical Analysis

Descriptive statistics are shown as mean and standard deviation. Parametric tests (*t*-student, correlations, and mediations) were used to conduct all analyses, as indicated by the central limit theorem for sample sizes over 500 participants (Lumley et al., 2002). Simultaneously, a Q-Q plot (quantile-quantile plot) was used for checking normality visually. Neither interaction by sex nor by age was observed, thereby all analyses are presented together for boys and girls and adolescents between 10 and 14 years old. However, Table 1 (participants’ characteristics) gives information about boys, girls, and all together to have a global vision of the study participants.

**Table 1:** Participants’ characteristics.

	*n*	All Mean ± SD	*n*	Boys Mean ± SD	*n*	Girls Mean ± SD	*p*-value
Age (years)	1,196	11.71, 1.06	606	11.68, 1.05	590	11.74, 1.08	0.312
Weight (kg)	1,183	50.28, 11.91	603	49.38, 11.99	580	51.21, 11.76	**0.002**
Height (cm)	1,183	152.41, 9.24	603	152.24, 10.19	580	152.57, 8.13	0.206
PHV	1,183	−0.55, 1.21	603	−1.28, 0.93	580	0.21, 0.98	**<0.001**
BMIz	1,183	1.04, 1.07	603	1.07, 1.10	580	1.01, 1.03	0.244
4SKF (mm)	1,150	64.56, 27.47	587	57.05, 23.77	563	72.40, 28.87	**<0.001**
WHtR	1154	0.46, 0.06	587	0.46, 0.06	567	0.45, 0.06	**<0.001**
SVI	1,196	56.08, 35.12	606	57.61, 34.16	590	54.51, 36.04	0.128
Cognitive performance	1,196	100.02, 4.85	606	99.70, 4.88	590	100.34, 4.80	**0.021**
Global fitness score	912	0.01, 3.10	460	−0.01, 3.21	452	0.03, 2.98	0.845
CRF (*z*score)	967	0.00, 1.00	491	0.00, 1.00	476	0.00, 1.00	0.602
MF (*z*score)	975	0.02, 1.68	489	0.03, 1.73	486	0.02, 1.62	0.960
SAF (*z*score)	976	0.00, 1.00	492	0.00, 1.00	484	0.00, 1.00	0.689

*SD, standard deviation; PHV, peak high velocity; BMIz, body mass index (zscore); 4SKF, sum-of-4-skinfolds; WHtR, waist-to-height ratio; SVI, school vulnerability index; CRF, cardiorespiratory fitness; MF, muscular fitness; SAF, speed-agility fitness. Bold values indicate statistical significance*.

The t-Student test was performed to compare boys’ and girls’ characteristics. Associations among fatness indicators, fitness components, and cognitive performance were performed by Pearson correlations (continuous variables) and Kendall’s tau-b to SVI (categorical variable). Moreover, multicollinearity was checked before performing the mediation analysis. Considering the high rate of participation and representativeness, missing data were not imputed.

**Figure 1:**
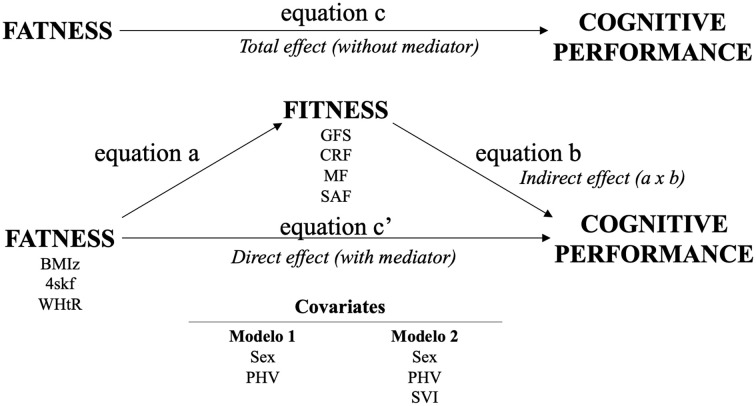
Theoretical approach and general mediation model. BMIz, body mass index (zscore); 4SKF, sum-of-4-skinfolds; WHtR, waist-to-height ratio; GFS, global fitness score; CRF, cardiorespiratory fitness; MF, muscular fitness; SAF, speed-agility fitness; PHV, peak high velocity; SVI, school vulnerability index.

The mediation model is presented in Figure 1. Overall, 24 mediation analyses were performed considering predictors (BMIz, 4SKF or WHtR), mediators (global fitness, CRF, MF, or SAF), outcome (cognitive performance), and two models (covariates). The general mediation model was structured as follows: equation (a) consisted of the predictor by the mediator; equation (b) was defined as a mediator by the outcome; equation (c) consisted of the predictor by the outcome; and finally, equation (c’) consisted of predictor and mediator by the outcome. Bias was reduced, adjusting analyses to relevant covariates; thus, two models were performed to test our objectives and hypotheses. Model 1: adjusted for sex and PHV, and model 2: adjusted for sex, PHV, and SVI. Note that: (a) the models were not adjusted for other fitness components to facilitate comparison between the percentage of mediation with the GFS (composed by the sum of the three fitness components); and (b) sample size by analysis changes depending on if participants have measures of all their variables (fatness, fitness, cognitive and covariates). Detailed sample size by analysis is presented as Supplementary Material (Supplementary Tables 1–3).

To evaluate the mediation effect, bootstrapping with 5,000 samples linear regression analysis was performed (Preacher and Hayes, 2008) through PROCESS SPSS script (Hayes, 2013). The indirect effect was considered significant if zero was outside the 95% confidence interval (Field, 2013). Percentage of mediation was estimated as 1– (equation c’ / equation c). Finally, the mediation was classified according to Nitzl et al. (Nitzl et al., 2016) as: (a) “Indirect-only” (Full mediation): the indirect effect only exists through the mediator, this means the indirect effect exists, but no direct effect; (b) “Complementary” (Partial mediation): a portion of the effect of the predictor on the outcome variable is mediated through the mediator, whereas predictor still explains a portion of outcome variable that is independent of a mediator, that means that the indirect and direct effect exists and point in the same direction; (c) “Competitive” (Partial mediation): the same as the complementary classification, both the indirect and direct effect exists but point in different directions; (d) “Direct-only” (No mediation): the direct effect exists, but no indirect effect; and (e) “No effect” (No mediation): neither direct and indirect effect exists (Zhao et al., 2010). For all analyses, the significance level was set at *p* <0.05.

## Results

Table 1 displays participants characteristics and differences by sex (boys 50.7% and girls 49.3%). Statistical differences in weight, PHV, WHtR, 4SKF and cognitive performance were found.

The correlation matrix among fatness indicators (i.e., BMIz, 4SKF, WHtR), fitness indicators (i.e., GFS, CRF, MF and SAF), SVI and cognitive performance are presented in Table 2. Overall, all physical fitness variables were negatively correlated with fatness indicators. In contrast, physical fitness variables were positively associated with cognitive performance, and fatness variables were negatively related to cognitive performance. Moreover, all correlations were statistically significant.

**Table 2:** Correlation matrix among fatness, fitness, and cognition variables.

	BMIz	4SKF	WHtR	GFS	CRF	MF	SAF	SVI
4SKF	**0.730**		
WHtR	**0.830**	**0.724**		
GFS	**−0.425**	**−0.475**	**−0.467**		
CRF	**−0.341**	**−0.399**	**−0.349**	**0.789**	
MF	**−0.458**	**−0.496**	**−0.511**	**0.906**	**0.558**	
SAF	**−0.208**	**−0.236**	**−0.235**	**0.785**	**0.496**	**0.547**
SVI	**0.089**	**0.054**	**0.126**	**−0.160**	**−0.139**	**−0.184**	**−0.054**
Cognitive performance	**−0.102**	**−0.094**	**−0.102**	**0.133**	**0.124**	**0.094**	**0.109**	−**0.146**

*BMIz, body mass index standard deviation; 4SKF, sum 4 skinfolds; WHtR, waist-to-height ratio; GFS, global fitness score; CRF, cardiorespiratory fitness; MF, muscular fitness; SAF, speed-agility fitness; SVI, school vulnerability index. Bold values indicate statistical significance*.

Table 3 shows a summary of all study mediations. Overall, it is possible to observe the variation in each meditation percentage and classification according to model 1 and 2. For BMIz: both CRF and SAF presented a “complementary” mediation (model 1) which changed to “Indirect only” mediation in model 2. The final mediation percentages (model 2) were 22.0% (SAF) and 29.0% (CRF). GFS and MF did not have any mediation effect after controlling for SVI (model 2). In 4SKF: the global fitness and all its components did not mediate the association between 4SKF and cognitive performance after controlling the analysis for SVI (model 2). For WHtR: GFS, CRF, and SAF presented a full mediation effect (“Indirect only”) in both models. The final mediation percentages (model 2) were 31.9% (SAF), 38.6% (CRF), and 54.8% (GFS). MF did not have any mediation effect. A complete description of all mediation analyses is presented as Supplementary Material (Supplementary Tables 1–3).

**Table 3:** Findings’ summary concerning the direct and indirect effect according to both models.

	BMIz	4SKF	WHtR
GFS	43.5%* → 36.9% = **Δ −6.6** Indirect only → No effect	**40.8% → 31.8%** = **Δ −9.0** No effect → No effect	51.8%* → 54.8* = **Δ +3.0** Indirect only → Indirect only
CRF	32.5%* → 29.0%* = **Δ −3.5** Complementary → Indirect only	**34.8% → 29.2%** = **Δ −5.6** Direct only → No effect	36.4%* → 38.6%* = **Δ +2.2** Indirect only → Indirect only
MF	**26.3% → 9.7%** = **Δ −16.6** Direct only → No effect	**23.0% → 3.0%** = **Δ −20.0** Direct only → Direct only	**31.8% → 19.1% Δ −12.7** No effect → No effect
SAF	19.7%* → 22.0%* = **Δ +2.3** Complementary → Indirect only	**19.0% → 19.7%** = **Δ +0.7** Direct only → Direct only	23.1%* → 31.9%* = **Δ +8.8** Indirect only → Indirect only

General scheme: Model 1 → Model 2 = Variation on mediation (Δ %); Model 1: Adjusted for sex and PHV; Model 2: Adjusted Model 1 + SVI. * indicates the indirect effect is statistically significant. 4SKF, sum-of-4-skinfolds; BMIz, Body Mass Index *z*-score; WHtR, Waist-to-Height Ratio; GFS, global fitness score; CRF, Cardiorespiratory Fitness; MF, Muscular Fitness; SAF, Speed-Agility Fitness. Mediation classifications: “Indirect-only” (Full mediation): the indirect effect only exists through the mediator, which means the indirect effect exists, but no direct effect; “Complementary” (mediation): indirect and direct effect exists and point in the same direction; “Indirect-only” (mediation): indirect effect exists, but no direct effect; “Direct-only” (non-mediation): direct effect exists, but no indirect effect; and “No effect” (non-mediation): neither direct and indirect effect exists. Percentage of mediation and classification.

## Discussion

This study aimed to determine the mediation role of a GFS and its components on the association between different fatness indicators related to fat distribution and a global cognitive score in adolescents and exploring the influence of SVI. Concerning the mediations, first, the influence of physical fitness as a mediator was modified according to what kind of fatness indicator related to fat distribution was analyzed; second, the GFS, CRF, and SAF showed a significant mediation role, whereas MF did not; and third, the SVI inclusion in the second model tends to modify the percentage and mediation’s classification; nonetheless, the favorable fitness role in the association between WHtR and cognitive performance seems to not be affected.

### Differences in Fitness Component Mediations

To date, the positive association between physical fitness and cognition is well-established in the literature (Donnelly et al., 2016). However, it is crucial in this research area to expand the exploratory approach to multiples fitness components due to the personal preferences of type of exercise and physical activities (which could improve a particular fitness component more than others), and also due to the differential association between each fitness component with cognitive skills. Regarding the latter, a study in children with overweight and obesity showed a positive association between MF and planning ability, between SAF and cognitive flexibility and inhibition, and finally CRF and a GFS with indicators of cognitive flexibility (Mora-Gonzalez et al., 2019). Another study based on the same sample of the present study showed that CRF, MF and SAF differed in their significant association according to the cognitive domain studied (cognitive flexibility, working memory, inhibitory control, or intelligence; Solis-Urra et al., 2021). In this sense, our findings showed that the GFS, CRF, and SAF were positively associated with the global cognitive score and mediated the relation between fatness and cognition. However, MF did not mediate any association.

These fitness component differences could be explained to a certain extent by the physiological influence of each one of them on brain indicators and also mechanisms related to fat oxidation. On the one hand, activities with a high CRF demand seem to activate the prefrontal cortex and the hippocampus and increase neurotrophins related to neurogenesis and angiogenesis at the cerebral level (Best, 2010). Indeed, children having a high CRF show greater total gray and white matter volume (Cadenas-Sanchez et al., 2020). This positive association between CRF and brain indicators also was observed in MF, SAF, and GFS. MF has been less studied, being its influence on brain functioning related to a direct neuromuscular mechanism boosting the strength and power demand, which seems to be dependent on the muscular contraction type (Yao et al., 2016; Solis-Urra et al., 2021). However, the MF influence in a whole-brain volumetric approach in children seems to not be independent of CRF (Esteban-Cornejo et al., 2017, 2021). In contrast, SAF involved speed of movements, agility, coordination, and a mix between power, strength and aerobic capacity which elicit high cognitive demands (Best, 2010; van der Fels et al., 2015). Hence, this study speculates that all differences mentioned could affect the variation in the mediations found in our study.

On the other hand, these fitness components in children and adolescents are developed through a diversity of physical activities (games, sports, physical education classes, active commuting, etc.) being difficult to isolate the development of a particular fitness component; however, there is a certain specificity in some physical activities. Thus, in addition to activating diverse brain zones to execute complex motor movements (Best, 2010), physical activities with higher intensity and duration can significantly increase the energy demand, which, in turn, improves fat oxidation (Chang et al., 2021), unlike strength exercises which have shown to be less effective (Chang et al., 2021). Thereby, recommendations to increase physical activity focusing on physical fitness improvement in children and adolescents are crucial to enhance their cognitive functioning through the direct influence of exercise and physical activity and indirectly, reducing the detrimental impact of fat on health.

### Fatness Indicators Related to Fat Distribution

Our findings reinforce the relevance of studying fatness indicators related to its distribution, showing that the fitness mediator role depends on the fatness indicator studied. Overall, excess adiposity in children and adolescents has been linked to higher inflammatory markers, which in turn, is a risk factor for neurodegeneration and cognitive impairment (Trollor et al., 2012; Caminiti et al., 2016). However, the three fatness indicators used in this study, which involved non-specific (BMIz), peripheral (4SKF), and central body fat distribution markers (WHtR), differ in their level of association with respect to the low-grade inflammation, becoming more pronounced as age advances (Cabral et al., 2019a,b). Thus, BMIz must be considered as a surrogate marker for obesity which does not differentiate between peripheral and central obesity, while between 4SKF and WHtR, the latter seems to be more related to inflammation and cognitive functioning (Caminiti et al., 2016).

In this sense, our results support the notion that physical fitness’ mediation role, mainly CRF and SAF, was more sensitive to WHtR followed by BMIz (no mediation with 4SKF). On one hand, metanalytic studies have concluded that interventions including exercise showed to be more effective in reducing visceral adiposity, regardless of the total body weight loss (Verheggen et al., 2016); and in children and adolescents with obesity, it was also observed that exercise is more effective than diet alone or than the combination between diet and exercise to reduce visceral fat (Verheggen et al., 2016; Vissers et al., 2016). On the other hand, a network meta-analysis showed that high-intensity interval training and aerobic exercise (between 30–60 min duration/session) were the most effective strategies to reduce visceral fat compared to strength exercise (Chang et al., 2021). Hence, this study speculates that, first, the mediation effect found in BMIz could be a consequence of the central fat included in this marker; and second, children and adolescents who accumulated more physical activities show increased CRF, SAF, and the GFS, which would consequently enhance the efficacy to oxidase and reduce visceral fat (Perez-Bey et al., 2020) and, in turn, influence cognition.

### Exploring SVI

A novel approach of the present study was to explore an indicator of vulnerability due to their implication in fatness, fitness and cognitive outcomes in children and adolescents (Jiménez-Pavón et al., 2010; Ursache and Noble, 2016a). Our findings show that SVI has a differentiated influence according to the model analyzed. For BMIz, SVI reduced all mediation effects except for SAF. However, CRF and SAF mediator role kept their statistical significance (29% and 22%, respectively). In the case of WHtR, a different scenario was present because the SVI inclusion increased the mediation role of the GFS, CRF, and SAF (Δ 3.0%, Δ 2.2%, and Δ 8.8%, respectively). All of them kept their statistical significance. Thus, on the one hand, these findings support our hypothesis showing the higher WHtR’s specificity compared to 4SKF and BMIz; and on the other hand, they support CRF and SAF’s relevant role regardless of adolescents’ school vulnerability influence.

The aforementioned is valuable to public health policy and educative communities, allowing to reduce the cognitive gap associated with children’s social background and obesity. Therefore, physical activity and physical fitness programs could be an efficient and low-cost strategy to improve cognition performance in children and adolescents, reducing the detrimental impact of fatness indicators and school vulnerability.

### Strengths and Limitations

A strength of this study is its large sample size of adolescents from a Latin-American country. This study has also included a full assessment of several fatness and fitness indicators that increase understanding in this research area. This study has used a robust cognitive score calculated by eight different tasks. Finally, exploring the influence of a vulnerability score allows this study to establish a novel finding with respect to the mediator role of fitness regardless of the adolescents’ social background profile. Nonetheless, this study has some limitations; first, its cross-sectional design implies that the cause-effect relationships among variables cannot be determined. Second, physical fitness was evaluated by field-based tests due to the high costs of gold-standard methods. Lastly, fatness was evaluated by a double indirect method, which could increase methodological biases. However, both fitness and fatness measures are feasible to implement in school settings.

### Conclusion

It is concluded that a higher level of physical fitness, mainly CRF and SAF, would mediate the detrimental influence of fatness on adolescents’ cognitive performance. The fatness indicator related to central fat distribution seemed to be more consistent, both theoretically and statistically. Finally, the mediator role of physical fitness in adolescents’ cognitive performance remains constant even in the presence of an important school vulnerability indicator, highlighting that it might play a relevant protective social role. Thereby, public health and educational strategies promoting physical fitness improvement through a wide diversity of physical activities are a determining factor in the reduction of the cognitive gap caused by obesity and social vulnerability in children and adolescents.

## Data Availability Statement

The raw data supporting the conclusions of this article will be made available by the authors, without undue reservation.

## Ethics Statement

The studies involving human participants were reviewed and approved by Bioethics and Biosafety Committee of the Pontificia Universidad Católica de Valparaíso (BIOEPUCV-H103-2016). Written informed consent to participate in this study was provided by the participants’ legal guardian/next of kin.

## Author Contributions

CC-M contributed to the design of the project and is the corresponding author. CC-M and SH-J conceptualized the design of the study. SH-J and CC-M analyzed the data and wrote the concept version of the manuscript. JS-M, PS-U, IE-C, JC-P, KS, NA-F, and GF critically reviewed the manuscript and edited the article. All authors have given final approval of the manuscript and agreed to be accountable for the accuracy and integrity of any part of the work. All authors contributed to the article and approved the submitted version.

## Conflict of Interest

The authors declare that the research was conducted in the absence of any commercial or financial relationships that could be construed as a potential conflict of interest.

## Publisher’s Note

All claims expressed in this article are solely those of the authors and do not necessarily represent those of their affiliated organizations, or those of the publisher, the editors and the reviewers. Any product that may be evaluated in this article, or claim that may be made by its manufacturer, is not guaranteed or endorsed by the publisher.

## References

[B1] Åberg M. A. I. Pedersen N. L. Torén K. Svartengren M. Bäckstrand B. Johnsson T.. (2009). Cardiovascular fitness is associated with cognition in young adulthood. Proc. Natl. Acad. Sci. U S A 106, 20906–20911. 10.1073/pnas.0905307106 19948959 PMC2785721

[B2] Best J. R. (2010). Effects of physical activity on children’s executive function: contributions of experimental research on aerobic exercise. Dev. Rev. 30, 331–351. 10.1016/j.dr.2010.08.001 21818169 PMC3147174

[B4] Brito N. H. Noble K. G. (2014). Socioeconomic status and structural brain development. Front. Neurosci. 8:276. 10.3389/fnins.2014.00276 25249931 PMC4155174

[B5] Brown T. T. Kuperman J. M. Chung Y. Erhart M. McCabe C. Hagler D. J.. (2012). Neuroanatomical assessment of biological maturity. Curr. Biol. 22, 1693–1698. 10.1016/j.cub.2012.07.002 22902750 PMC3461087

[B6] Cabral M. Bangdiwala S. I. Severo M. Guimarães J. T. Nogueira L. Ramos E. (2019a). Central and peripheral body fat distribution: different associations with low-grade inflammation in young adults. Nutr. Metab. Cardiovasc. Dis. 29, 931–938. 10.1016/j.numecd.2019.05.066 31303476

[B7] Cabral M. Severo M. Barros H. Guimarães J. T. Ramos E. (2019b). Longitudinal association of adiposity and high-sensitivity C-reactive protein from adolescence into early adulthood. Nutr. Metab. Cardiovasc. Dis. 29, 590–597. 10.1016/j.numecd.2019.03.008 31078361

[B8] Cadenas-Sanchez C. Migueles J. H. Erickson K. I. Esteban-Cornejo I. Catena A. Ortega F. B. (2020). Do fitter kids have bigger brains. Scand. J. Med. Sci. Sports 30, 2498–2502. 10.1111/sms.13824 33314403

[B9] Caminiti C. Armeno M. Mazza C. S. (2016). Waist-to-height ratio as a marker of low-grade inflammation in obese children and adolescents. J. Pediatr. Endocrinol. Metab. 29, 543–551. 10.1515/jpem-2014-0526 26887032

[B10] Chang Y.-H. Yang H.-Y. Shun S.-C. (2021). Effect of exercise intervention dosage on reducing visceral adipose tissue: a systematic review and network meta-analysis of randomized controlled trials. Int. J. Obes. 45, 982–997. 10.1038/s41366-021-00767-9 33558643

[B11] Cristi-Montero C. Courel-Ibáñez J. Ortega F. B. Castro-Piñero J. Santaliestra-Pasias A. Polito A.. (2019). Mediation role of cardiorespiratory fitness on the association between fatness and cardiometabolic risk in european adolescents: the HELENA study. J. Sport Health Sci. 10, 360–367. 10.1016/j.jshs.2019.08.003 33993922 PMC8167318

[B12] de Onis M. Onyango A. W. Borghi E. Siyam A. Nishida C. Siekmann J. (2007). Development of a WHO growth reference for school-aged children and adolescents. Bull. World Health Organ. 85, 660–667. 10.2471/blt.07.043497 18026621 PMC2636412

[B13] Diamond A. Ling D. S. (2016). Conclusions about interventions, programs and approaches for improving executive functions that appear justified and those that, despite much hype, do not. Dev. Cogn. Neurosci. 18, 34–48. 10.1016/j.dcn.2015.11.005 26749076 PMC5108631

[B14] Donnelly J. E. Hillman C. H. Castelli D. Etnier J. L. Lee S. Tomporowski P.. (2016). Physical activity, fitness, cognitive function and academic achievement in children: a systematic review. Med. Sci. Sports Exerc. 48, 1197–1222. 10.1249/MSS.0000000000000901 27182986 PMC4874515

[B15] Esteban-Cornejo I. Reilly J. Ortega F. B. Matusik P. Mazur A. Erhardt E.. (2020). Paediatric obesity and brain functioning: the role of physical activity—A novel and important expert opinion of the european childhood obesity group. Pediatr. Obes. 15:e12649. 10.1111/ijpo.12649 32459068

[B16] Esteban-Cornejo I. Cadenas-Sanchez C. Contreras-Rodriguez O. Verdejo-Roman J. Mora-Gonzalez J. Migueles J. H.. (2017). A whole brain volumetric approach in overweight/obese children: Examining the association with different physical fitness components and academic performance. the activebrains project. Neuroimage 159, 346–354. 10.1016/j.neuroimage.2017.08.011 28789992

[B17] Esteban-Cornejo I. Stillman C. M. Rodriguez-Ayllon M. Kramer A. F. Hillman C. H. Catena A.. (2021). Physical fitness, hippocampal functional connectivity and academic performance in children with overweight/obesity: the activebrains project. Brain Behav. Immun. 91, 284–295. 10.1016/j.bbi.2020.10.006 33049365

[B18] Feinstein L. Bynner J. (2004). The importance of cognitive development in middle childhood for adulthood socioeconomic status, mental health and problem behavior. Child Dev. 75, 1329–1339. 10.1111/j.1467-8624.2004.00743.x 15369517

[B19] Field A. (2013). Discovering Statistics Using IBM SPSS Statistics. Sussex, UK: SAGE Publications Ltd. 954 pp.

[B20] Flores-Mendoza C. Mansur-Alves M. Ardila R. Rosas R. D. Guerrero-Leiva M. K. Maqueo M. E. L.-G.. (2015). Fluid intelligence and school performance and its relationship with social variables in Latin american samples. Intelligence 49, 66–83. 10.1016/j.intell.2014.12.005

[B21] Hackman D. A. Farah M. J. (2009). Socioeconomic status and the developing brain. Trends Cogn. Sci. 13, 65–73. 10.1016/j.tics.2008.11.003 19135405 PMC3575682

[B22] Halfon N. Larson K. Slusser W. (2013). Associations between obesity and comorbid mental health, developmental and physical health conditions in a nationally representative sample of us children aged 10 to 17. Acad. Pediatr. 13, 6–13. 10.1016/j.acap.2012.10.007 23200634

[B3] Hayes A. F. (2013). Introduction to Mediation, Moderation, and Conditional Process Analysis: A Regression-Based Approach. New York, NY: The Guilford Press.

[B23] Herting M. M. Chu X. (2017). Exercise, cognition and the adolescent brain. Birth Defects Res. 109, 1672–1679. 10.1002/bdr2.1178 29251839 PMC5973814

[B24] Jiménez-Pavón D. Ortega F. B. Ruiz J. R. Chillón P. Castillo R. Artero E. G.. (2010). Influence of socioeconomic factors on fitness and fatness in Spanish adolescents: the AVENA study. Int. J. Pediatr. Obes. 5, 467–473. 10.3109/17477160903576093 20233152

[B25] Kao S.-C. Westfall D. R. Parks A. C. Pontifex M. B. Hillman C. H. (2017). Muscular and aerobic fitness, working memory and academic achievement in children. Med. Sci. Sports Exerc. 49, 500–508. 10.1249/MSS.0000000000001132 27776002

[B26] López V. Oyanedel J. C. Bilbao M. Torres J. Oyarzún D. Morales M.. (2017). School achievement and performance in chilean high schools: the mediating role of subjective wellbeing in school-related evaluations. Front. Psychol. 8:1189. 10.3389/fpsyg.2017.01189 28769838 PMC5509788

[B27] Lemes V. Gaya A. R. Sadarangani K. P. Aguilar-Farias N. Rodriguez-Rodriguez F. Martins CM de L.. (2021). Physical fitness plays a crucial mediator role in relationships among personal, social and lifestyle factors with adolescents’ cognitive performance in a structural equation model the cogni-action project. Front. Pediatr. 9:656916. 10.3389/fped.2021.656916 34195161 PMC8236613

[B28] Liang J. Matheson B. E. Kaye W. H. Boutelle K. N. (2014). Neurocognitive correlates of obesity and obesity-related behaviors in children and adolescents. Int. J. Obes. (Lond) 38, 494–506. 10.1038/ijo.2013.142 23913029 PMC4456183

[B29] Lloyd R. S. Oliver J. L. Faigenbaum A. D. Myer G. D. De Ste Croix M. B. A. (2014). Chronological age vs. biological maturation: implications for exercise programming in youth. J. Strength Cond. Res. 28, 1454–1464. 10.1519/JSC.0000000000000391 24476778

[B30] Lumley T. Diehr P. Emerson S. Chen L. (2002). The importance of the normality assumption in large public health data sets. Annu. Rev. Public Health 23, 151–169. 10.1146/annurev.publhealth.23.100901.140546 11910059

[B31] Moore S. A. McKay H. A. Macdonald H. Nettlefold L. Baxter-Jones A. D. G. Cameron N.. (2015). Enhancing a somatic maturity prediction model. Med. Sci. Sports Exerc. 47, 1755–1764. 10.1249/MSS.0000000000000588 25423445

[B32] Mora-Gonzalez J. Esteban-Cornejo I. Cadenas-Sanchez C. Migueles J. H. Molina-Garcia P. Rodriguez-Ayllon M.. (2019). Physical fitness, physical activity and the executive function in children with overweight and obesity. J. Pediatr. 208, 50–56.e1. 10.1016/j.jpeds.2018.12.028 30902422

[B33] Morrison G. E. Simone C. M. Ng N. F. Hardy J. L. (2015). Reliability and validity of the neurocognitive performance test, a web-based neuropsychological assessment. Front. Psychol. 6:1652. 10.3389/fpsyg.2015.01652 26579035 PMC4630791

[B34] Nitzl C. Roldan J. L. Cepeda G. (2016). Mediation analysis in partial least squares path modeling: helping researchers discuss more sophisticated models. Ind. Manag. Data Syst. 116, 1849–1864. 10.1108/imds-07-2015-0302

[B35] Perez-Bey A. Ruiz J. R. Ortega F. B. Martinez-Gomez D. Mota J. Veiga O. L.. (2020). Bidirectional associations between fitness and fatness in youth: a longitudinal study. Scand. J. Med. Sci. Sports 30, 1483–1496. 10.1111/sms.13684 32297361

[B36] Preacher K. J. Hayes A. F. (2008). Asymptotic and resampling strategies for assessing and comparing indirect effects in multiple mediator models. Behav. Res. Methods 40, 879–891. 10.3758/brm.40.3.879 18697684

[B37] Reuter C. P. Brand C. Silveira JF de C. Schneiders L de B. Renner J. D. P. Borfe L.. (2021). Reciprocal longitudinal relationship between fitness, fatness and metabolic syndrome in brazilian children and adolescents: a 3-year longitudinal study. Pediatr. Exerc. Sci. 33, 74–81. 10.1123/pes.2020-0197 33857920

[B38] Ruiz J. R. Castro-Piñero J. España-Romero V. Artero E. G. Ortega F. B. Cuenca M. M.. (2011). Field-based fitness assessment in young people: the ALPHA health-related fitness test battery for children and adolescents. Br. J. Sports Med. 45, 518–524. 10.1136/bjsm.2010.075341 20961915

[B39] Ruiz-Hermosa A. Mota J. Díez-Fernández A. Martínez-Vizcaíno V. Redondo-Tébar A. Sánchez-López M. (2019). Relationship between weight status and cognition in children: a mediation analysis of physical fitness components. J. Sports Sci. 38, 13–20. 10.1080/02640414.2019.1676538 31597515

[B40] Sahoo K. Sahoo B. Choudhury A. K. Sofi N. Y. Kumar R. Bhadoria A. S. (2015). Childhood obesity: causes and consequences. J. Family Med. Prim. Care 4, 187–192. 10.4103/2249-4863.154628 25949965 PMC4408699

[B41] Sawyer S. M. Azzopardi P. S. Wickremarathne D. Patton G. C. (2018). The age of adolescence. Lancet Child Adolesc. Health 2, 223–228. 10.1016/S2352-4642(18)30022-1 30169257

[B42] Schoentgen B. Gagliardi G. Défontaines B. (2020). Environmental and cognitive enrichment in childhood as protective factors in the adult and aging brain. Front. Psychol. 11:1814. 10.3389/fpsyg.2020.01814 32793081 PMC7385286

[B43] Schwartz D. H. Leonard G. Perron M. Richer L. Syme C. Veillette S.. (2013). Visceral fat is associated with lower executive functioning in adolescents. Int. J. Obes. 37, 1336–1343. 10.1038/ijo.2013.104 23797144 PMC5061567

[B44] Solis-Urra P. Olivares-Arancibia J. Suarez-Cadenas E. Sanchez-Martinez J. Rodríguez-Rodríguez F. Ortega F. B.. (2019). Study protocol and rationale of the “Cogni-action project” a cross-sectional and randomized controlled trial about physical activity, brain health, cognition and educational achievement in schoolchildren. BMC Pediatr. 19:260. 10.1186/s12887-019-1639-8 31349791 PMC6659252

[B45] Solis-Urra P. Sanchez-Martinez J. Olivares-Arancibia J. Castro Piñero J. Sadarangani K. P. de Moraes Ferarri G. L.. (2021). Physical fitness and its association with cognitive performance in chilean schoolchildren: the cogni-action project. Scand. J. Med. Sci. Sports 31, 1352–1362. 10.1111/sms.13945 33638920

[B46] Stillman C. M. Esteban-Cornejo I. Brown B. Bender C. M. Erickson K. I. (2020). Effects of exercise on brain and cognition across age groups and health states. Trends Neurosci. 43, 533–543. 10.1016/j.tins.2020.04.010 32409017 PMC9068803

[B47] Tomkinson G. R. Lang J. J. Léger L. A. Olds T. S. Ortega F. B. Ruiz J. R.. (2019). Response to criticisms of the 20 m shuttle run test: deflections, distortions and distractions. Br. J. Sports Med. 53, 1200–1201. 10.1136/bjsports-2018-100348 30728128

[B48] Trollor J. N. Smith E. Agars E. Kuan S. A. Baune B. T. Campbell L.. (2012). The association between systemic inflammation and cognitive performance in the elderly: the sydney memory and ageing study. Age (Dordr) 34, 1295–1308. 10.1007/s11357-011-9301-x 21853262 PMC3448981

[B49] Ursache A. Noble K. G. (2016a). Neurocognitive development in socioeconomic context: multiple mechanisms and implications for measuring socioeconomic status. Psychophysiology 53, 71–82. 10.1111/psyp.12547 26681619 PMC4685721

[B50] Ursache A. Noble K. G. (2016b). Socioeconomic status, white matter and executive function in children. Brain Behav. 6:e00531. 10.1002/brb3.531 27781144 PMC5064342

[B51] van der Fels I. M. J. te Wierike S. C. M. Hartman E. Elferink-Gemser M. T. Smith J. Visscher C. (2015). The relationship between motor skills and cognitive skills in 4–16 year old typically developing children: a systematic review. J. Sci. Med. Sport 18, 697–703. 10.1016/j.jsams.2014.09.007 25311901

[B52] Vazquez C. E. Cubbin C. (2020). Socioeconomic status and childhood obesity: a review of literature from the past decade to inform intervention research. Curr. Obes. Rep. 9, 562–570. 10.1007/s13679-020-00400-2 32785878

[B53] Verheggen R. J. H. M. Maessen M. F. H. Green D. J. Hermus A. R. M. M. Hopman M. T. E. Thijssen D. H. T. (2016). A systematic review and meta-analysis on the effects of exercise training versus hypocaloric diet: distinct effects on body weight and visceral adipose tissue. Obes. Rev. 17, 664–690. 10.1111/obr.12406 27213481

[B54] Vissers D. Hens W. Hansen D. Taeymans J. (2016). The effect of diet or exercise on visceral adipose tissue in overweight youth. Med. Sci. Sports Exerc. 48, 1415–1424. 10.1249/MSS.0000000000000888 27314412

[B55] von Elm E. Altman D. G. Egger M. Pocock S. J. Gøtzsche P. C. Vandenbroucke J. P.. (2008). The strengthening the reporting of observational studies in epidemiology (STROBE) statement: guidelines for reporting observational studies. J. Clin. Epidemiol. 61, 344–349. 10.1016/j.jclinepi.2007.11.008 18313558

[B56] Yang X. Telama R. Leskinen E. Mansikkaniemi K. Viikari J. Raitakari O. T. (2007). Testing a model of physical activity and obesity tracking from youth to adulthood: the cardiovascular risk in young Finns study. Int. J. Obes. 31, 521–527. 10.1038/sj.ijo.0803459 16953253

[B57] Yao W. X. Jiang Z. Li J. Jiang C. Franlin C. G. Lancaster J. L.. (2016). Brain functional connectivity is different during voluntary concentric and eccentric muscle contraction. Front. Physiol. 7:521. 10.3389/fphys.2016.00521 27895590 PMC5108928

[B58] Zhao X. Lynch J. G. Chen Q. (2010). JD served as editor and GF served as associate editor for this. reconsidering baron and kenny: myths and truths about mediation analysis. J. Consumer Res. 37, 197–206. 10.1086/651257

