# Supplementary material for: Mediation Role of Physical Fitness and Its Components on the Association Between Distribution-Related Fat Indicators and Adolescents’ Cognitive Performance: Exploring the Influence of School Vulnerability. The Cogni-Action Project
Source: Front Behav Neurosci. 2021 Sep 8;15:746197. doi: 10.3389/fnbeh.2021.746197 (PMC8456005; doi:10.3389/fnbeh.2021.746197)
Supplement: Supplementary file 1 [file Table_1.docx]

**Table S1.** Mediation analysis between BMIz and cognitive performance mediate by physical fitness.

|  |  | n | Predictor - Mediator | Mediator – Outcome | Total effect | Direct effect | Indirect effect | Mediation | Mediation |
| --- | --- | --- | --- | --- | --- | --- | --- | --- | --- |
|  |  |  | a | b | c | c' | a x b | % | Type |
| BMIz-GFS-Cogn | Model 1 | 912 | -1.24* (-1.41, -1.07) | 0.17* (0.06, 0.28) | -0.47* (-0.77, -0.18) | -0.27 (-0.59, 0.06) | -0.21* (-0.35, -0.07) | 43.5% | (FM)Indirect only |
|  | Model 2 | 912 | -1.17* (-1.34, -1.00) | 0.11* (0.00, 0.23) | -0.36* (-0.66, -0.07) | -0.23 (-0.55, 0.09) | -0.13 (-0.27, 0.00) | 36.9% | (NM) No effect |
| BMIz-CRF-Cogn | Model 1 | 962 | -0.32* (-0.37, -0.26) | 0.48* (0.16, 0.80) | -0.47* (-0.75, -0.19) | -0.32* (-0.62, -0.02) | -0.15* (-0.27, -0.05) | 32.5% | (PM)Complementary |
|  | Model 2 | 962 | -0.30* (-0.36, -0.25) | 0.35* (0.03, 0.67) | -0.36* (-0.65, -0.08) | -0.26 (-0.56, 0.04) | -0.11* (-0.21, -0.01) | 29.0% | (FM)Indirect only |
| BMIz-MF-Cogn | Model 1 | 975 | -0.72* (-0.81, -0.63) | 0.17 (-0.03, 0.37) | -0.46* (-0.75, -0.18) | -0.34* (-0.66, -0.02) | -0.12 (-0.26, 0.02) | 26.3% | (NM)Direct only |
|  | Model 2 | 975 | -0.68* (-0.77, -0.59) | 0.05 (-0.16, 0.25) | -0.34* (-0.62, -0.06) | -0.31 (-0.62, 0.01) | -0.03 (-0.17, 0.11) | 9.7% | (NM)No effect |
| BMIz-SAF-Cogn | Model 1 | 970 | -0.19* (-0.25, -0.14) | 0.45* (0.14, 0.76) | -0.44* (-0.73, -0.16) | -0.36* (-0.65, -0.07) | -0.09* (-0.16, -0.03) | 19.7% | (PM)Complementary |
|  | Model 2 | 970 | -0.19* (-0.24, -0.13) | 0.39* (0.08, 0.69) | -0.33* (-0.61, -0.04) | -0.26 (-0.54, 0.03) | -0.07* (-0.14, -0.02) | 22.0% | (FM)Indirect only |

*BMIz: Body Mass Index (zscore); CRF: Cardiorespiratory Fitness; MF: Muscular Fitness; SAF: Speed-Agility Fitness; GFS: Global Fitness Score; Cogn: Cognition; SVI: School Vulnerability Index. Model 1: Adjusted for sex and PHV; Model 2: Adjusted Model 1 + SVI. *p-value <0.05; FM: full mediation; NM: no mediation; PM: partial mediation.*
