# Supplementary material for: Mediation Role of Physical Fitness and Its Components on the Association Between Distribution-Related Fat Indicators and Adolescents’ Cognitive Performance: Exploring the Influence of School Vulnerability. The Cogni-Action Project
Source: Front Behav Neurosci. 2021 Sep 8;15:746197. doi: 10.3389/fnbeh.2021.746197 (PMC8456005; doi:10.3389/fnbeh.2021.746197)
Supplement: Supplementary file 2 [file Table_2.docx]

**Table S2.** Mediation analysis between 4skf and cognitive performance mediate by physical fitness.

|  |  | n | Predictor - Mediator | Mediator – Outcome | Total effect | Direct effect | Indirect effect | Mediation | Mediation |
| --- | --- | --- | --- | --- | --- | --- | --- | --- | --- |
|  |  |  | a | b | c | c' | a x b | % | Type |
| 4SKF-GFS-Cogn | Model 1 | 887 | -0.06* (-0.07, -0.05) | 0.15* (0.03, 0.27) | -0.02* (-0.03, -0.01) | -0.01 (-0.03, 0.00) | -0.01 (-0.02, 0.00) | 40.8% | (NM)No effect |
|  | Model 2 | 887 | -0.06* (-0.06, -0.05) | 0.10 (-0.02, 0.21) | -0.02* (-0.03, -0.01) | -0.01 (-0.03, 0.00) | -0.01 (-0.01, 0.00) | 31.8% | (NM)No effect |
| 4SKF-CRF-Cogn | Model 1 | 936 | -0.02* (-0.02, -0.01) | 0.45* (0.12, 0.79) | -0.02* (-0.03, -0.01) | -0.01* (-0.03, -0.00) | -0.01 (-0.01, 0.00) | 34.8% | (NM)Direct only |
|  | Model 2 | 936 | -0.02* (-0.02, -0.01) | 0.32 (-0.02, 0.66) | -0.02* (-0.03, -0.01) | -0.01 (-0.03, 0.00) | 0.00 (-0.01, 0.00) | 29.2% | (NM)No effect |
| 4SKF-MF-Cogn | Model 1 | 949 | -0.03* (-0.04, -0.03) | 0.14 (-0.07, 0.35) | -0.02* (-0.03, -0.01) | -0.02* (-0.03, -0.00) | 0.00 (-0.01, 0.00) | 23.0% | (NM)Direct only |
|  | Model 2 | 949 | -0.03* (-0.04, -0.03) | 0.02 (-0.19, 0.23) | -0.02* (-0.03, 0.00) | -0.02* (-0.03, -0.00) | 0.00 (-0.01, 0.01) | 3.0% | (NM)Direct only |
| 4SKF-SAF-Cogn | Model 1 | 944 | -0.01* (-0.01, -0.01) | 0.40* (0.09, 0.72) | -0.02* (-0.03, -0.01) | -0.02* (-0.03, -0.01) | 0.00 (-0.01, 0.00) | 19.0% | (NM)Direct only |
|  | Model 2 | 944 | -0.01* (-0.01, -0.01) | 0.34* (0.03, 0.65) | -0.02* (-0.03, 0.00) | -0.01* (-0.03, -0.00) | 0.00 (-0.01, 0.00) | 19.7% | (NM)Direct only |

*4SKF: sum-4-skinfolds; CRF: Cardiorespiratory Fitness; MF: Muscular Fitness; SAF: Speed-Agility Fitness; GFS: Global Fitness Score; Cogn: Cognition; SVI: School Vulnerability Index. Model 1: Adjusted for sex and PHV; Model 2: Adjusted Model 1 + SVI. *p-value <0.05; NM: no mediation.*
