# Supplementary material for: Mediation Role of Physical Fitness and Its Components on the Association Between Distribution-Related Fat Indicators and Adolescents’ Cognitive Performance: Exploring the Influence of School Vulnerability. The Cogni-Action Project
Source: Front Behav Neurosci. 2021 Sep 8;15:746197. doi: 10.3389/fnbeh.2021.746197 (PMC8456005; doi:10.3389/fnbeh.2021.746197)
Supplement: Supplementary file 3 [file Table_3.docx]

**Table S3.** Mediation analysis between WHtR and cognitive performance mediate by physical fitness.

|  |  | n | Predictor - Mediator | Mediator – Outcome | Total effect | Direct effect | Indirect effect | Mediation | Mediation |
| --- | --- | --- | --- | --- | --- | --- | --- | --- | --- |
|  |  |  | a | b | c | c' | a x b | % | Type |
| WHtR-GFS-Cogn | Model 1 | 892 | -26.66* (-30.00, -23.33) | 0.17* (0.06, 0.29) | -8.92* (-14.75, -3.09) | -4.30 (-10.86, 2.26) | -4.62* (-7.83, -1.64) | 51.8% | (FM)Indirect only |
|  | Model 2 | 892 | -25.17* (-28.52, -21.81) | 0.13* (0.01, 0.24) | -5.86* (-11.70, -0.01) | -2.65 (-9.15, 3.85) | -3.21* (-6.15, -0.32) | 54.8% | (FM)Indirect only |
| WHtR-CRF-Cogn | Model 1 | 941 | -6.46* (-7.56, -5.35) | 0.51* (0.18, 0.84) | -9.03* (-14.72, -3.33) | -5.74 (-11.79, 0.32) | -3.29* (-5.57, -1.17) | 36.4% | (FM)Indirect only |
|  | Model 2 | 941 | -6.01* (-7.13, -4.89) | 0.39* (0.06, 0.72) | -6.08* (-11.79, -0.38) | -3.74 (-9.76, 2.29) | -2.35* (-4.42, -0.37) | 38.6% | (FM)Indirect only |
| WHtR-MF-Cogn | Model 1 | 954 | -15.91* (-17.60, -14.21) | 0.18 (-0.04, 0.39) | -8.83* (-14.50, -3.15) | -6.02 (-12.63, 0.58) | -2.80 (-6.12, 0.59) | 31.8% | (NM)No effect |
|  | Model 2 | 954 | -15.05* (-16.75, -13.34) | 0.07 (-0.14, 0.28) | -5.56 (-11.24, 0.11) | -4.50 (-11.01, 2.01) | -1.06 (-4.20, 2.15) | 19.1% | (NM)No effect |
| WHtR-SAF-Cogn | Model 1 | 949 | -4.31* (-5.46, -3.17) | 0.44* (0.12, 0.75) | -8.2* (-13.86, -2.53) | -6.30 (-12.11, -0.49) | -1.89* (-3.42, -0.57) | 23.1% | (FM)Indirect only |
|  | Model 2 | 949 | -4.13* (-5.30, -2.97) | 0.39* (0.08, 0.70) | -5.05 (-10.71, 0.62) | -3.44 (-9.23, 2.35) | -1.61* (-3.12, -0.32) | 31.9% | (FM)Indirect only |

*WHtR: Waist-to-Height Ratio; CRF: Cardiorespiratory Fitness; MF: Muscular Fitness; SAF: Speed-Agility Fitness; GFS: Global Fitness Score; Cogn: Cognition; SVI: School Vulnerability Index. Model 1: Adjusted for sex and PHV; Model 2: Adjusted Model 1 + SVI. *p-value <0.05; FM: full mediation; NM: no mediation.*
