# Supplementary figures and images for: Mediation Role of Physical Fitness and Its Components on the Association Between Distribution-Related Fat Indicators and Adolescents’ Cognitive Performance: Exploring the Influence of School Vulnerability. The Cogni-Action Project
Source: Front Behav Neurosci. 2021 Sep 8;15:746197. doi: 10.3389/fnbeh.2021.746197 (PMC8456005; doi:10.3389/fnbeh.2021.746197)

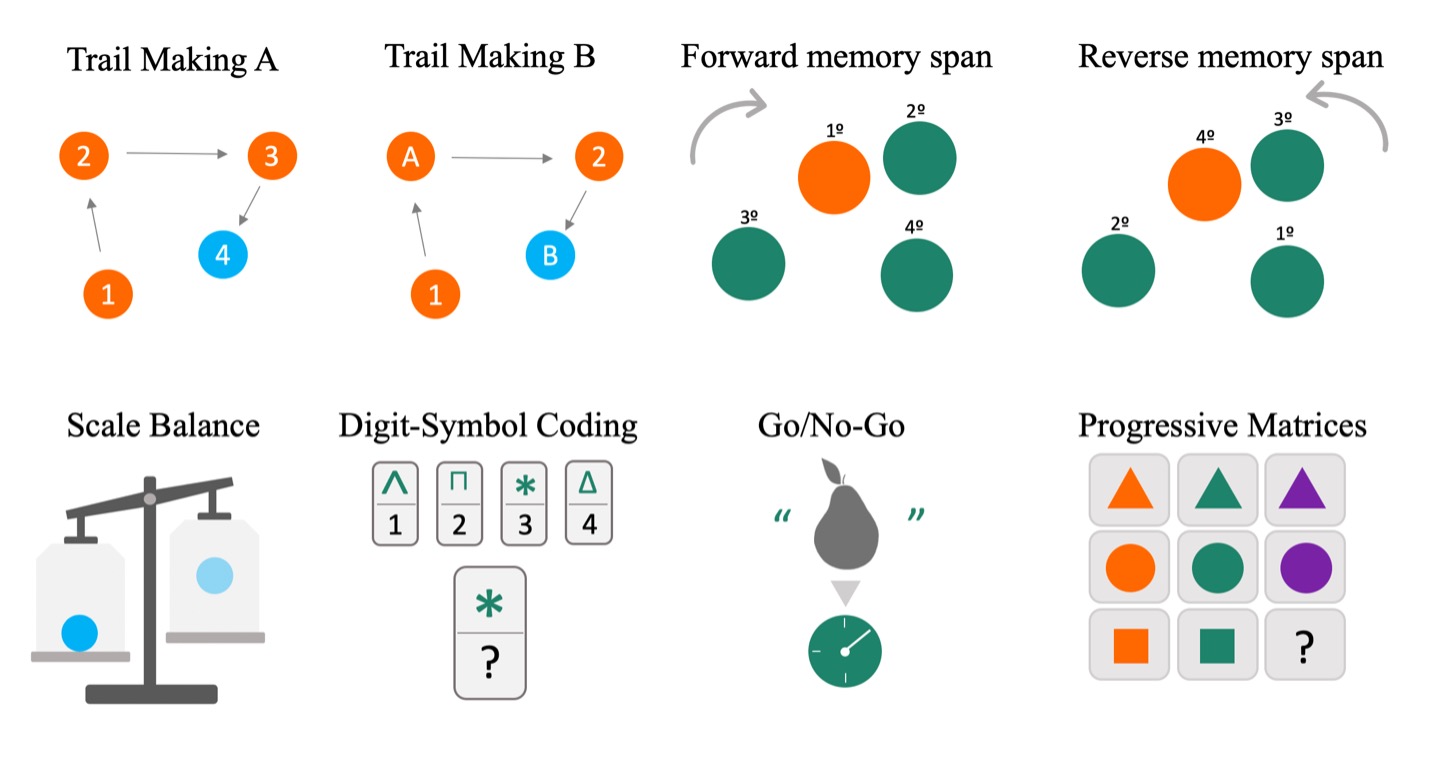

Supplement: Supplementary file 4 [file Image_1.jpg]
